# Advance Care Planning for Adolescents and Young Adults with Cancer: A Clinical Review

**DOI:** 10.3390/children13101405

**Published:** 2026-10-14

**Authors:** Joshua Gorlick, Swapna Shanmugiavelayutham, Jill Ann Jarrell, Claire Crawford, Joshua Roy, Rachel M. Kim, Anastasia Deeter, Stephanie Fetzko, Matthew T. McEvoy

**Affiliations:** 1 Division of Pediatrics, Baylor College of Medicine, Houston, TX 77030, USA; jajarrel@texaschildrens.org (J.A.J.); jxroy@texaschildrens.org (J.R.); rachel.kim@bcm.edu (R.M.K.); slfetzko@texaschildrens.org (S.F.);; 2 Division of Pediatric Hematology/Oncology, Texas Children’s Hospital, Houston, TX 77030, USA; aldeeter@texaschildrens.org; 3 Division of Palliative Care, Texas Children’s Hospital, Houston, TX 77030, USA

**Keywords:** advance care planning, adolescent and young adults, cancer/oncology, serious illness communication, goal-concordant care, psychosocial care

## Abstract

**Highlights:**

**What are the main findings?**

• Developmentally tailored, family-centered advance care planning (ACP) interventions improve engagement, decision-making, and psychosocial outcomes for adolescents and young adults (AYAs) with cancer.
• Specialty palliative care teams add valuable expertise to AYA advance care planning, but any oncology provider can initiate these conversations using practical, available tools.

**What are the implications of the main finding?**

• Advance care planning should be tailored to AYA patients’ evolving autonomy, identity, and family dynamics, and revisited throughout the illness course.
• Oncology teams should not wait for palliative care referral to begin advance care planning, while institutions work to expand equitable access to specialty palliative care.

**Abstract:**

Adolescents and young adults (AYAs) diagnosed with cancer experience unique psychosocial distress and worse clinical outcomes compared to their younger and older peers. Additionally, the developmental emergence of identity and independence for AYAs—and the effects of oncology therapy on these milestones—adds complexity to communication and medical decision-making among patients and families. Given these circumstances, AYAs require thoughtful approaches for advance care planning (ACP). In many cases, specialty palliative care (SPC) teams can provide an extra layer of support to both patients and families as well as primary oncology teams in navigating ACP and ensuring goal-concordant care. While some adult studies have recently questioned the effectiveness of standard ACP methods, other new investigations specifically in the AYA cancer population have demonstrated multifaceted benefits. With the understudied nature of both ACP techniques and the AYA population in general and the constant evolution of communication preferences in the digital technology era, pediatric oncology clinicians may be unsure of current best practices for how to guide their older patients during conversations about their illness. We aim to review the most recent evidence regarding ACP for AYAs with cancer, with an emphasis on describing practical bedside tools and the advantages of collaborating with SPC teams to promote optimal outcomes.

## 1. Introduction

A cancer diagnosis during adolescence and young adulthood can carry not only difficult clinical experiences but also profound developmental consequences, disrupting identity formation, autonomy, and the pursuit of goals that define this period of life. In many cases, the dichotomous frameworks of pediatric and adult medical care insufficiently address these concerns [1,2]. Furthermore, adolescents and young adults (AYAs, ages 15–39) with cancer face worse survival outcomes than both younger children and older adults for many malignancies, lending particular urgency to advance care planning (ACP). ACP refers to the process of eliciting and documenting a patient’s personal goals, values, and preferences for medical care in the setting of serious illness [3,4,5,6,7]. ACP can be understood as an umbrella term describing a progressive process of ongoing communication of the patient’s preferences and goals, which informs shared decision-making, translating into documentation when appropriate (e.g., advance directives, code status orders), and ultimately supporting goal-concordant care throughout the illness course. The AYA population as defined here (ages 15–39) spans markedly different developmental, legal, and psychosocial stages, from minors under parental authority to fully autonomous adults; the approaches and tools discussed throughout this review are intended to be developmentally tailored rather than applied uniformly across this range [7].

ACP for AYAs is a topic of considerable clinical and research interest, but best practices have yet to be determined due to the nuances and complexities of both the patient population and clinical models [6,7,8]. Standard ACP approaches were largely developed for older adults and may not adequately account for the distinct needs of younger patients who are simultaneously navigating individuation, evolving parental relationships, and the legal transition to autonomous decision-making [9]. The unique communication preferences in this age group are also being rapidly reshaped by digital technology and social media, adding further complexity to an already challenging clinical task [10,11]. Furthermore, clinician-level barriers also contribute to inconsistent ACP implementation: many oncology providers report limited awareness of ACP tools or emotional reservations about engaging in such discussions with families [8,12]. Finally, the mechanics of ACP are difficult to study, and its presumed benefits are difficult to prove—academic shortcomings that might affect providers’ level of clinical engagement [13]. Indeed, in adult medicine, some leaders have questioned the utility of broad ACP approaches, and occasional studies demonstrate neutral or negative outcomes [14,15,16]. Nonetheless, for AYAs with cancer, a growing body of evidence, including randomized prospective studies, suggests that developmentally tailored and family-centered interventions can yield meaningful benefits [17,18,19,20,21].

Given the complexity in navigating ACP conversations, specialty palliative care (SPC) teams are well-positioned to support AYAs, their families, and primary oncology teams, with specific expertise spanning goals-of-care communication, psychosocial support, and end-of-life (EOL) planning [5,6,7]. However, the authors emphasize that ACP conversations may be conducted by a wide range of healthcare providers as part of routine oncology care, and SPC involvement is not a prerequisite for initiating them. Furthermore, many locations (e.g., rural areas or low-and-middle-income countries) lack dedicated SPC resources [22,23]. In any practice setting, valuable ACP tools designed for or adaptable to AYA patients are readily available, offering clinicians concrete resources across a range of clinical contexts and patient preferences [24,25,26,27].

To improve ACP for AYAs at our large academic center, an evidence-based institutional clinical practice guideline was developed in 2024 by physician faculty in both oncology and palliative care and was subsequently reviewed and approved by divisional leadership for clinical implementation. For this article, an updated literature review was completed, followed by additional input from multidisciplinary faculty with diverse practice experiences. As this is a clinical review, the literature discussed here was identified primarily through PubMed (searches conducted through June 2026) using terms related to pediatric and AYA oncology advance care planning, and was supplemented by the clinical and research expertise of our multidisciplinary author team; articles were selected for topical relevance and clinical applicability. Here, we review the most current evidence regarding ACP for AYAs with cancer seen in pediatric oncology settings. We begin by describing the unique developmental, structural, and communication challenges that define oncology care in this population. We then examine current evidence and approaches for conducting ACP conversations with AYAs. Next, we discuss how SPC teams can complement primary oncology teams in supporting goal-concordant care. Finally, we highlight practical, bedside-ready tools available to patients, caregivers, and clinicians alike.

## 2. Unique Considerations for Oncology Care in AYAs

Adolescents and young adults with cancer face a dual burden: the biological and medical challenges of their disease, and the profound disruption of a developmental period already defined by transition and uncertainty. AYAs have concerns about identity formation, body image, social status, gaining independence in relation to parents, and making decisions about future goals such as career, education, and family [1,6,7,28]. A cancer diagnosis during this period can complicate the cultivation of self-confidence, individuality, and self-sufficiency [29]. Because many AYAs are treated in clinical settings focused on younger (pediatric) or older (adult) counterparts, their routine concerns can easily evolve into distinct unmet psychosocial needs that affect their care [2]. These developmental and social dynamics for AYAs can heavily influence their health-related goals, and clinicians should be mindful of these factors in their ACP approaches.

AYAs with cancer also encounter structural barriers rooted in social determinants of health and the design of healthcare systems that can significantly impact outcomes. AYAs have a higher likelihood of lacking health insurance, placing them at elevated risk of economic burden and limited access to care [30]. Uninsured and underinsured AYAs are more likely to present with advanced disease and experience worse cancer-specific mortality compared to privately insured peers [30,31]. Many adolescent patients diagnosed within a pediatric system face the added disruption of transitioning out of that setting upon reaching institutional age thresholds, which may coincide with disease relapse or vulnerable periods following frontline therapy. Aging out of insurance plans or institutions can cause delays in care, treatment adherence issues, subsequent clinical decline, and reduced eligibility for clinical trial enrollment [32,33]. Moreover, the AYA population comprises patients who experience a discordance between their age category (i.e., child or adult as defined by health systems) and their disease (as defined by standard therapy availability and provider comfort level in management). This reality not only affects individual clinical care [34], but also the ability for disease knowledge advancement through research, as only recently have clinical trials and oncology societies started to bridge the historical dichotomy between children and adults [35,36]. These compounding susceptibilities contribute to a well-documented survival gap for many patients aged 15–39 and can create unique challenges in treatment planning [3,4,35].

Some AYAs with advanced disease experience additional age-related clinical limitations. Many AYAs with relapsed or refractory cancer seek enrollment in early-phase clinical trials [37]. Because these studies enroll small numbers of patients and are often available only at specialized centers, many AYAs must travel long distances or may not have access because of strict age eligibility criteria. Another challenge for patients in the United States is the ability to pursue concurrent care—defined as receiving both disease-directed and hospice-based care over the same period—due to the link between hospice services and insurance. Unlike those in many countries with universal healthcare, AYAs > 21 years old living in the U.S. must forego disease-directed therapy to enroll in hospice [38]. Even for younger AYAs eligible for concurrent care under federal legislation (e.g., the Concurrent Care for Children Requirement established via the Affordable Care Act), the current state of pediatric hospice care in the U.S. suffers from limited resources, inconsistent implementation, and inequitable access [39,40,41,42]. These realities demonstrate the complexity of treatment planning for AYAs with advanced disease and the need for individualized ACP approaches.

Influenced by their social development and clinical setting, AYA patients have distinct communication preferences [28]. In studies about oncologic communication broadly, AYAs and their parents—like virtually all patients—desire compassion, hopefulness, and honesty from their clinicians, while AYAs place added import on providers keeping a calm demeanor [43]. Notably, a majority of AYAs report desiring full prognostic information, and this preference tends to increase over the illness course [11]. Some oncologists may limit prognostic transparency out of concern for undermining hope, yet evidence suggests this concern is largely unfounded: while serious illness communication should be calibrated to individual preferences, thorough prognostic disclosure can coexist with and even reinforce hope, even when the likelihood of cure is low [28,44,45]. Even for “low-stakes” conversations during routine longitudinal care, clinicians should be mindful that AYA patients often utilize digital platforms (e.g., social media) for illness-related information and peer support, and consider how such sources may affect their care [10,46]. Finally, as patients move through adolescence into adulthood, the legal and interpersonal landscape of decision-making affects communication approaches, with those under age 18 typically lacking the legal authority to independently consent to treatment, even as they prefer to be informed and involved in their own care [6,7,28]. Consistent with this desire for involvement in medical decisions, a study measuring decisional control preferences among AYAs with cancer and other complex medical conditions found that most patients preferred active collaborative (39%) or shared (34%) decision-making roles rather than passive ones, with oncology patients trending somewhat more passive than their non-oncology peers and preferences becoming more active over time since diagnosis [47]. Conversely, some patients > 18 years old may, for various reasons, defer to their parents’ decision-making, an ethically and legally supportable position. Together, these distinctive elements for AYAs underlay the importance of applying thoughtful and evidence-based approaches for ACP.

## 3. Approaching Advance Care Planning for AYAs

The authors acknowledge that terms like “advance care planning” and “goals of care” may feel nebulous, and applaud efforts to work toward a multidisciplinary consensus definition [48,49]. The foremost task for ACP includes eliciting the patient’s personal goals, values, and preferences related to their future medical care. The primary aim of ACP is to support patients and families through serious illness and ultimately align their wishes with subsequent care delivery, which is commonly deemed “goal-concordant care”. A recent Position Statement from the Canadian Paediatric Society provides a comprehensive guide for ACP as a multidimensional and iterative process [5]. Notably, the import of ACP elements has also been highlighted by the American Society of Clinical Oncology (ASCO) consensus guidelines for patient-clinician communication [50].

Discussions related to ACP and goals of care span the illness course and provider teams [51,52]. Given the gravity of a cancer diagnosis—regardless of its curability—for an AYA patient, the authors contend that ACP starts with early care conversations [53]. In fact, consistent high-quality communication during frontline therapy can help establish an open and trusting patient–provider relationship that, in addition to several intrinsic benefits, may be foundational for more difficult decisions in the future [28,45,54]. Topics related to ACP evolve over the disease trajectory, usually building upon information gathered from prior periods, and always involve asking open-ended questions, active listening, education, and anticipatory guidance (Table 1). In addition to the oncologist, other health professionals (e.g., advanced practice providers, social workers, nurses, psychologists, chaplains, palliative care providers) may develop relationships with the patient and family over time or simply gain an essential goal-related insight during a single encounter; multidisciplinary collaboration is key to ensure optimal outcomes [55].

Recent reviews have delineated the essential elements of ACP across all diseases for AYAs specifically [63,64,65]. While some practitioners may think of ACP as related solely to legal documentation tasks (Table 2), the authors propose and these reviews indicate that ACP encompasses much more than just advance directives or code status orders, and is distinct from, though related to, serious illness communication and goals-of-care discussions, which represent components of the broader ACP process. For example, Linane et al. identifies shared decision-making, optimal communication techniques, the need for individualization and flexibility, and timing of ACP initiation as additional core concepts [63]. Likewise, another recent review reinforces the need for providers to practice cultural humility and proactiveness in identifying AYAs’ preferences and times of clinical stability for raising discussion topics [7]. Regarding clinical communication and the role of parents in decision-making, one creative prospective study demonstrated that accurate prognostic understanding influences treatment-related goals [66]. Finally, unlike cases with younger children, clinicians for AYAs must navigate unique challenges involving the patient-caregiver-provider triad, including potential decision-making discordance, caregivers hoping to preserve their “good parent” beliefs, and mutual protectionism [28,67,68,69].

The potential benefits of ACP span multiple domains. For example, a recent systematic review for adult ACP evaluated completion of EOL discussions and advance directives (primary outcomes) as well as delivery of goal-concordant care, communication and education measures, patient satisfaction, healthcare utilization, and symptom control (secondary outcomes), finding that many ACP-related interventions improved outcomes [70]. A downstream effect of ACP, the intensity of EOL care has been shown to be high (and potentially inequitable or goal-discordant) in AYAs across diverse settings [71,72,73]. Given the unique circumstances of AYAs with cancer, more population-specific studies are urgently needed to address known issues such as long-term effects on family members, including grief and ACP-related parental decisional regret [7,74]. Furthermore, studies investigating ACP disparities in adults could be expanded or replicated for AYAs [75].

The available intervention studies in this population are heterogeneous in design, sample size, and outcome measures, and vary considerably in the strength of evidence they provide; feasibility and acceptability findings should be distinguished from patient-centered clinical outcomes, and their collective results should not be interpreted as uniform evidence of benefit. With several prospective studies measuring similar outcomes and some novel considerations, evidence-based insights related to ACP for AYAs with advanced cancer have grown over the past 15 years. For example, as part of the multi-center PRISM randomized clinical trial, one cohort (n = 26, ages 12–24) used the Voicing My Choices^TM^ (VMC) ACP tool in conjunction with stress management coaching and reported positive feedback regarding participation, demonstrating acceptability [18,24,76]. A similar trial evaluated the DECIDES intervention, a web-based tool with an optional supportive coach, (n = 53, ages 15–24). AYAs in both intervention arms reported higher decision-making involvement and lower decision regret compared to usual care [19]. Additionally, a recent mixed-methods study investigated a video-based decision aid in AYA-parent dyads (n = 85, AYA ages 18–39) and noted increased ACP reflection and learning led to changes in care preferences [20]. In Europe, the Belgian BOOST study enrolled adolescent-parent dyads (n = 49, AYA ages 10–18) and found that parents randomized to the intervention (four ACP sessions) had more positive attitudes toward ACP, though the authors ultimately felt their results were underpowered [21]. Finally, a multi-center prospective non-randomized study evaluating VMC demonstrated increased engagement in ACP conversations and decreased short-term anxiety (n = 149 AYAs, ages 18–39) [77].

The study that has generated the most outcome analyses to date for AYA ACP is the multi-center randomized FACE-TC trial, which included three weekly family-centered sessions using multiple ACP tools for the intervention group [17,25,27]. The first cohort of 30 AYA-caregiver dyads (ages 14–21) showed that increased ACP engagement was associated with increased patient-caregiver congruence for care preferences and increased education on EOL topics [17]. With an expanded cohort of 126 dyads (n = 83 for intervention group), subsequent reports have demonstrated multifaceted positive outcomes for those receiving the intervention, including insights into adolescents’ preferred timing of ACP discussions and spiritual well-being, increased completion of advance directives, and decreased patient anxiety at 3 months [78,79]; increased congruence with and leeway for caregivers/potential surrogates, sustained over 12 months, with some race/ethnicity disparities [80,81]; and increased positive caregiving appraisals without an increase in distress for family member participants [82]. However, not all outcomes have been beneficial: when investigating quality of life via symptom screening, intervention adolescents reported increases in anxiety, depression, and pain interference at 12 months, signaling the importance of recurring conversations to maintain optimal outcomes [83]. Taken together, these studies illustrate both the promise and the current limitations of AYA ACP intervention research: outcomes span feasibility, process, and patient-centered clinical domains, sample sizes are often small, and further follow-up and analyses will be needed to determine which approaches most reliably translate into goal-concordant care. Notably, outcomes such as engagement, communication, documentation completion, and caregiver congruence, while meaningful, are process-oriented measures and are not equivalent to demonstrating goal-concordant care, which remains inherently difficult to measure and is infrequently assessed directly in the current literature. Further follow-up and analyses from these studies will help shape best practices going forward.

Finally, the unique ACP dynamics have been understudied in two specific populations: childhood cancer survivors and AYA patients with children themselves. Survivors often endure late or long-term morbidities that may lead to post-cancer ACP needs when they become AYAs [84]. In addition to their personal cancer experience, survivors may have cognitive impairments, mental health concerns, or psychosocial considerations that affect their health-related goals [85,86,87]. Additionally, for older AYAs with cancer, having one’s own young children can affect willingness to engage in ACP, and many patients’ goals of care may involve ensuring adequate support for them [88,89]. More research is needed for these vulnerable groups.

## 4. Integrating Palliative Care

ACP is one of the core domains within palliative care, defined as specialized medical care that aims to provide holistic support for those with serious illness to promote quality of life [6,7,23]. Specialty palliative care (SPC) often includes dedicated interdisciplinary teams composed of physicians, nurses, social workers, and spiritual care providers. Of course, other domains—symptom management, psychosocial and spiritual care, EOL care, and grief/bereavement support—overlap substantially with ACP, and practitioners’ interventions and support often address multiple areas directly or indirectly. These domains and general palliative care principles have been extensively reviewed for AYAs elsewhere [6,7]. The fundamentals of such care can be provided by virtually any provider (doing so is often termed primary palliative care), and many interdisciplinary oncology teams provide extraordinary palliative care to their patients. However, complex or difficult cases or under-resourced oncology settings may benefit from SPC expertise and collaboration. Notably, thoughtful efforts to ensure clear roles based on variable practice models are often warranted [90,91,92,93].

In the last few decades, the multi-dimensional benefits of (early) involvement of an SPC team have become well-established for both children and adults with cancer [94,95]. Given the pediatric/adult dichotomy in practice settings, most SPC research does not investigate AYAs specifically. Given that high-quality AYA-specific evidence remains limited, some recommendations discussed below are extrapolated from pediatric and adult oncology populations and should therefore be interpreted cautiously. However, multiple high-quality prospective studies in adults demonstrate increased quality of life/well-being (achieved early and sustained over time, including as death approaches, and including when added concurrently to populations that already have built-in psychosocial support); improved symptom burden; improved mental health in patients and caregivers; more ACP discussions; less chemotherapy received near EOL; increased frequency of death occurring at home; and even increased survival in select populations [95,96,97,98,99]. Anecdotally, the authors have also seen benefits of an independent SPC team providing perspective-taking and assisting in communication and prognostication for patients with cancer [100].

With the potential benefits of palliative care, multiple formal society policies (e.g., ASCO) advocate for early integration as the standard of care within (pediatric) oncology [23,101,102,103,104]. Referral processes for SPC are generally better established for adults compared to children or adolescents [6,7,105,106]. Even where pediatric resources are available, many barriers to SPC involvement remain [107]. However, many studies have investigated avenues for overcoming such difficulties, including automatic consult systems, quality improvement initiatives, cross-specialty education (e.g., communication workshops) and debriefing (e.g., Schwartz rounds) collaborations, creation of embedded palliative oncology clinics, and shared multi-disciplinary tumor boards [107,108,109,110,111,112,113,114,115]. Ideally, when SPC teams follow AYAs with cancer, they can work together with oncology teams and offer expertise in all palliative care domains, building upon prior ACP discussions and then ensuring comfort, whole-family attention, and goal-concordant care through EOL and beyond [6,7,92]. As patient wishes often extend to how they are remembered after their death (including their family’s coping), SPC teams can be especially helpful with managing anticipatory grief, funeral planning, bereavement support, and education related to comprehensive EOL care [6,7,116,117,118,119,120].

## 5. Advance Care Planning Practical Tools

The landscape of ACP tools has expanded in recent years, offering interactive decision-making support and adaptive conversation prompts for patients, families, and clinicians (Table 3). The evidence supporting these tools varies considerably: a subset, such as Voicing My Choices™, has been evaluated in randomized or prospective AYA-specific trials, while most of the remaining resources are descriptive, consensus-based, or adapted from broader clinical practice rather than formally studied in this population. Beyond these curated resources, providers may work with AYAs creatively to provide ACP that connects to their unique interests. Aside from verbal discussion, many AYAs prefer interaction through music, art, games, animals, and play [121,122,123]. At our hospital, for example, one author followed an 18-year-old patient with advanced cancer who had difficulty engaging in traditional ACP approaches. The author discovered that the patient loved hot sauce and the “Hot Ones” YouTube show in which celebrities answer increasingly personal questions as they eat chicken wings with increasingly spicy sauces. During a subsequent visit, the author brought hot sauces and chips to the patient’s room and asked him questions from the VMC booklet as he tried each sauce. By the end of this “challenge,” he provided a complete description of his wishes for treatment and comfort. These imaginative approaches to ACP are often best performed in tandem with staff who know the patient well and via a less “medical” framework (e.g., art/music therapists, child life specialists, animal-assisted therapy coordinators) [117]. Notably, clinicians in these realms can affect ACP in both indirect and direct ways: a discussion might provide insight into a patient’s wishes that can be incorporated into their overall care plan, and the therapeutic interventions may help relieve symptoms (e.g., pain, anxiety) and stress in patients or caregivers that can act as a barrier to engaging in ACP conversations. Ultimately, thoughtful provider collaboration can lead to novel solutions to some of the challenges seen in AYA ACP.

## 6. Conclusions & Future Directions

Advance care planning for AYAs with cancer is a dynamic, developmentally responsive process that extends beyond discrete discussions and documentation of EOL preferences and reflects deep relationships between patients, family members, and interdisciplinary health professionals. As highlighted throughout this review, effective ACP for AYAs is longitudinal, iterative, and addresses the evolving values, priorities, and autonomy of AYA patients. One limitation of this review is its pediatric-oriented authorship, which may lead to elements of care for older AYAs being under-represented. A further limitation is that, while we have aimed to represent both favorable and unfavorable findings throughout, we cannot fully exclude the possibility of selection bias in the literature discussed. Looking forward, not only will cancer care continue evolving in the precision oncology and artificial intelligence era, but AYA perspectives and communication dynamics may change as well [136]. Such transformations may bring difficulties and also may aid in addressing ACP-related disparities. Ultimately, patient-centered, multidisciplinary ACP approaches, often involving an SPC team, can adapt with changing times to support decision-making, promote shared understanding, and ensure compassionate and goal-concordant care for those with advanced illness. This includes remaining attentive to parents’ own priorities in end-of-life care, honest communication, symptom relief, and preservation of the parent–child relationship, which can help guide oncology and SPC teams alike in supporting the whole family unit [137].

## Figures and Tables

**Table 1:** Overview of Advance Care Planning Topics Across the Illness Course.

Topic Categories	Time Period Diagnosis Relapse/Progression Nearing End of Life			Ref. *
Personal history	School, work, interests, activities, values, hopes, worries, fears	Any changes or new elements compared to prior, function and ability to work or attend school as health declines	Legacy making, digital memorials, * preferred location of death, prioritization of interests/remaining time	[56]
Support systems	Family, social groups, spirituality *	How are these support systems working for the patient?	Social visits (e.g., extended family members), religious rituals, funeral planning	[57]
Prognosis	Likelihood of cure, ensuring understanding	Salvageability, life expectancy	Life expectancy * with more precision	[58]
Disease-directed therapy	Options (if applicable; e.g., chemotherapy regimen A vs. B, type of local control surgery for limb sarcoma)	Relapse therapy options, early phase trials, second opinions, breaks * in therapy to focus on other goals	Therapy with different goals (e.g., slow tumor growth), preferred intensity/location of care	[59]
Therapy side effects	How to address expected symptoms (e.g., alopecia), fertility preservation *	New anticipated symptoms with new therapies, potential change in tolerance of symptoms	Palliative interventions with expected increased symptoms (e.g., from disease more than therapy)	[60]
Palliative care (PC)	Often primary PC via oncology team, early integration	Increased specialty PC involvement in many cases	Specialty PC or hospice may lead care, concurrent care (if eligible)	–
Legal documentation	Default surrogate or family member, informed consent for interventions	Introduce advance directives, affirm preferred surrogate, more complex consent * for early phase trials	Complete advance directives, confirm code status, documents to support home-based care	[61]
Communication preferences	How does the patient like to receive information (e.g., when, with whom, amount of detail) and make decisions (shared decision-making spectrum); utilization of language interpreter *; respect for identity and preferred titles			[62]

Note: This table is intended to describe stereotypical topics (i.e., oversimplified and not exhaustive), generally building upon one another and likely growing in complexity or intensity as disease progresses (from left to right). The topics are listed in columns for ease of reading; however, in actual clinical practice, there are many additional opportunities for ACP in between these points and providers should be proactive about exploring topics and be open to virtually any ACP topic at any time per patient preference. Additional references are provided for select topics (indicated by an asterisk) that are not already covered in the main text.

**Table 2:** Advance Directives and End of Life Care Documentation in the United States.

Document/Order	Ages	Purpose	Key Points
Health Care Proxy/Medical Power of Attorney (MPOA)	≥18 yo	Designates a surrogate decision-maker if the patient loses decision-making capacity.	Patients aged 18 years or older who can make and verbalize their medical wishes should complete this legal document. This form can also designate limitations to the surrogate’s decision-making authority.
Directive to Physicians and Surrogates (e.g., living will)	≥18 yo	Legal document that outlines a patient’s specific preferences for end-of-life care.	Can serve as a guide for a patient’s MPOA. It may include choices related to CPR, mechanical ventilation, tube feeding, pain management, and organ donation.
Physician [or Medical] Orders for Life-Sustaining Treatment (POLST/MOLST); Out-of-hospital DNR form	Any	Translates current treatment preferences into actionable medical orders. Often used by families to facilitate home-based care (e.g., family wishes for the patient not to receive resuscitative measures if death occurs at home).	Generally intended for patients with serious illness or limited life expectancy; complements rather than replaces an advance directive. For in-home death, this form protects families from unwanted CPR from paramedics, police involvement to investigate circumstances of death, and delays in acceptance of body by a funeral home. Caregivers maintain decision-making control and flexibility—they can choose not to show the form to paramedics if they wish for CPR to be attempted.
Withdrawal of Life Sustaining Treatment (WOLST)	Any	In the setting of incurable illness and organ failure, an active process of removing advanced technological care with anticipation of death occurring soon.	Indicates that goals of care emphasize comfort more than life-prolongation, though both can always be hoped for. Usually occurs in a hospital, though some hospices may be able to support such care in the home. For example, removing an endotracheal tube is often deemed “compassionate extubation.”
Code status (e.g., DNR)	Any	In medical settings, orders that document a patient’s preferences regarding CPR in the setting of cardiac arrest or respiratory failure as well as broader aspects of care in the setting of incurable illness.	Default order is Full Code (all eligible life-saving interventions). A DNR/DNI or DNAR order indicates the patient desires a “natural” death rather than attempts at CPR or intubation, but their overall goals of care and life expectancy can span a wide range. A No Escalation order is generally for patients anticipating end-of-life, though goals may still include life-prolongation and comfort, with the emphasis varying by individual. No Escalation is created in addition to DNI/DNR orders and means that the patient will continue their current level of care rather than increasing to higher intensity treatments.

Note: The table presents the current framework for terminology and legal documentation within the United States. There is considerable variability in the international community for ACP related terminology and legal documentation, which is beyond the scope of the current review. Abbreviations: CPR, cardiopulmonary resuscitation; DNAR, do not attempt resuscitation; DNR/DNI, Do Not Resuscitate/Do Not Intubate.

**Table 3:** Advance Care Planning Resources.

Resource	Description	Ref.
*Patient-Focused Tools*		
Voicing My Choices^TM^ (VMC)	Developed by a clinical interdisciplinary team with feedback from young adults with serious illness, this ACP guide uses accessible language and a combination of multiple-choice answers and written responses to help AYAs express their preferences for comfort, support, medical care, and how they wish to be remembered. VMC provides a place to document preferences such as choice of medical power of attorney and code status, but it is a communication tool rather than a legal document; patients age 18 or older must still complete a separate, state-specific legal advance directive for these designations to be binding.	[18,24,124,125]
Five Wishes^TM^	While not specifically designed for AYAs, this communication tool was created by the same organization as Voicing My Choices and may be preferable to some young adults. With easy-to-understand language, the booklet covers five domains, including (1) surrogate decision-making; (2) therapy interests and limitations; (3) symptom control and comfort; (4) goal-concordant care delivery; and (5) legacy/memory-based desires. In some locations, this tool can also act as a binding legal document. The digital version is available in English and Spanish, and the paper version is available in over 25 languages.	[17,25]
Go Wish^TM^	Produced by the non-profit Coda Alliance, Go Wish gamifies ACP, serving as an interactive communication tool that assists patients in expressing their care preferences. Some AYAs find it easier to select the most important components of their care in a card game rather than writing them down. Providers can take a photo of the selected cards to include wishes in the patient’s chart. The program also offers Pediatric Go Wish Together, a group card game for children and their caregivers. Cards are available in multiple languages, and published studies describe their implementation in diverse settings globally.	[26,126,127]
*Family Resources*		
Courageous Parents Network (CPN)	Created by a bereaved family, CPN provides support for families to help with medical decision-making for children with serious illnesses. Its ACP media library includes videos, printable guides, and blogs with perspectives from providers and caregivers to provide families with comprehensive tools that guide difficult conversations with their children. CPN also offers a companion guide for clinicians. Content is currently offered in English and Spanish.	[128,129]
PREPARE^TM^	Developed by Dr. Rebecca Sudore, a palliative care physician at UCSF, PREPARE is a free, interactive online program that uses videos and step-by-step guidance to help users choose a decision-maker, clarify what matters most, and prepare an advance directive. It is designed primarily for older adults with chronic illness but is also promoted for AYAs and can support state-specific advance directive completion. The core program is available in English and Spanish; state-specific advance directives are available in many additional languages.	[130]
The Conversation Project	A public engagement initiative of the Institute for Healthcare Improvement (IHI), this resource contains conversation guides for caregivers of children and adults to facilitate discussion about their loved one’s wishes. The guides offer tips for approaching these conversations, sample scripts, and reassurance for caregivers. Guides are available in English and several other languages.	[131]
*Provider Training Tools*		
VitalTalk^®^	Founded in the 1990s as the NCI-funded OncoTalk program, this clinician training resource teaches communication skills for serious illness conversations. Online modules and live courses include skills for delivering serious news, responding to emotion, discussing prognosis, and clarifying goals of care. Training materials are offered in English. Adaptations have been made by disease, provider specialty, patient population, and social culture.	[112,132,133]
Ariadne Labs	A joint initiative between Brigham and Women’s Hospital and Harvard School of Public Health, this program offers The Serious Illness Conversation Guide, which provides questions for clinicians to use to ensure that care aligns with patients’ goals, values, and preferences. The core guide is available in English; related COVID-19-era conversation guides are available in several languages including Spanish and Vietnamese.	[134]
Respecting Choices^®^	Developed by Gundersen Health System in La Crosse, Wisconsin, this facilitator-driven ACP model trains both clinicians and non-clinicians to guide structured conversations tailored to a patient’s place in the illness trajectory, from early planning for healthy adults to in-the-moment decisions for patients who are actively dying. Programs are organized through a certification pathway and have been adapted for pediatric and AYA populations at some health systems. Materials are available in English and Spanish.	[17,27]
Center to Advance Palliative Care (CAPC)	Based at the Icahn School of Medicine at Mount Sinai, CAPC is a national membership organization supporting the development and growth of palliative care programs. Its online curriculum offers training modules with free CE/CME credit for clinicians to build clinical communication skills in serious illness conversations with their patients. While the focus is primarily on adult patients and their caregivers, many topics apply to ACP for AYAs and caregivers. Organizations may purchase annual memberships to provide all staff with access. Training materials are offered in English, with select patient/family-facing handouts also available in Spanish.	[135]

Abbreviations: ACP, advance care planning; AYA, adolescent and young adult; CAPC, Center to Advance Palliative Care; CE/CME, continuing education/continuing medical education; COVID, Coronavirus Disease-19; CPN, Courageous Parents Network; NCI, National Cancer Institute (U.S.); UCSF, University of California-San Francisco; VMC, Voicing My Choices. Note: The categories for this table are intended for ease of reading and are not restrictive; many resources can be utilized by patients, family members, and providers alike.

## Data Availability

No new data were created or analyzed in this study. Data sharing is not applicable to this article.
